# Tetra­kis[μ-2-(3,4-dimeth­oxy­phen­yl)acetato]-κ^4^ *O*:*O*′;κ^3^ *O*,*O*′:*O*;κ^3^ *O*:*O*,*O*′-bis­{[2-(3,4-dimeth­oxy­phen­yl)acetato-κ^2^ *O*,*O*′](1,10-phenanthroline-κ^2^ *N*,*N*′)samarium(III)}

**DOI:** 10.1107/S1600536810037153

**Published:** 2010-10-09

**Authors:** Jia-Lu Liu, Hua-Qiong Li, Guo-Liang Zhao

**Affiliations:** a College of Chemistry and Life Sciences, Zhejiang Normal University, Jinhua 321004, People’s Republic of China, and, Zhejiang Normal University Xingzhi College, Jinhua 321004, People’s Republic of China

## Abstract

In the centrosymmetric dinuclear title complex, [Sm_2_(C_10_H_11_O_4_)_6_(C_12_H_8_N_2_)_2_], the Sm^III^ ion is nine-coordinated by seven O atoms of five 2-(3,4-dimeth­oxy­phen­yl)acetate (DMPA) ligands and two N atoms of one bis-chelating 1,10-phenanthroline (phen) ligand, forming a distorted tricapped trigonal-prismatic environment. The DMPA ligands coordinate in bis-chelate, bridging and bridging tridentate modes. An intra­molecular C—H⋯O hydrogen bond occurs. Inter­molecular C—H⋯O inter­actions are also present in the crystal.

## Related literature

For the importance of coordination chemistry in magnetism, see: Fang & Zhang (2006 ▶); Yao *et al.* (2008 ▶); Wang & Sevov (2008 ▶). For related structures, see: Li *et al.* (2008 ▶); Liu *et al.* (2010 ▶). 
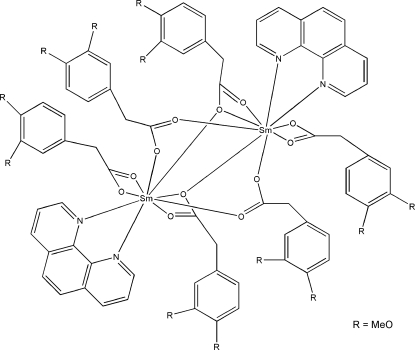


## Experimental

### 

#### Crystal data

[Sm_2_(C_10_H_11_O_4_)_6_(C_12_H_8_N_2_)_2_]
*M* *_r_* = 1832.26
Triclinic, 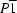

*a* = 12.3696 (1) Å
*b* = 12.4344 (1) Å
*c* = 14.7467 (1) Å
α = 90.641 (1)°
β = 103.492 (1)°
γ = 116.648 (1)°
*V* = 1953.71 (3) Å^3^
*Z* = 1
Mo *K*α radiation
μ = 1.57 mm^−1^
*T* = 296 K
0.35 × 0.15 × 0.09 mm

#### Data collection

Bruker APEXII area-detector diffractometer
Absorption correction: multi-scan (*SADABS*; Bruker, 2009 ▶) *T*_min_ = 0.757, *T*_max_ = 0.874
30687 measured reflections
9023 independent reflections
7540 reflections with *I* > 2σ(*I*)
*R*_int_ = 0.032

#### Refinement

*R*[*F* ^2^ > 2σ(*F* ^2^)] = 0.031
*wR*(*F* ^2^) = 0.102
*S* = 0.76
9023 reflections
514 parameters
H-atom parameters constrained
Δρ_max_ = 0.80 e Å^−3^
Δρ_min_ = −0.49 e Å^−3^

### 

Data collection: *APEX2* (Bruker, 2009 ▶); cell refinement: *SAINT* (Bruker, 2009 ▶); data reduction: *SAINT*; program(s) used to solve structure: *SHELXS97* (Sheldrick, 2008 ▶); program(s) used to refine structure: *SHELXL97* (Sheldrick, 2008 ▶); molecular graphics: *SHELXTL* (Sheldrick, 2008 ▶); software used to prepare material for publication: *SHELXL97*.

## Supplementary Material

Crystal structure: contains datablocks I, global. DOI: 10.1107/S1600536810037153/fk2022sup1.cif

Structure factors: contains datablocks I. DOI: 10.1107/S1600536810037153/fk2022Isup2.hkl

Additional supplementary materials:  crystallographic information; 3D view; checkCIF report

## Figures and Tables

**Table 1:** Hydrogen-bond geometry (Å, °)

*D*—H⋯*A*	*D*—H	H⋯*A*	*D*⋯*A*	*D*—H⋯*A*
C1—H1*A*⋯O5^i^	0.96	2.54	3.323 (5)	139
C21—H21*C*⋯O4^ii^	0.96	2.36	3.274 (5)	160
C23—H23*A*⋯O4^ii^	0.93	2.52	3.404 (4)	160
C31—H31*A*⋯O3	0.93	2.55	3.040 (5)	113

Symmetry codes: (i) 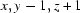; (ii) 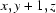.

**Fig. 1.**
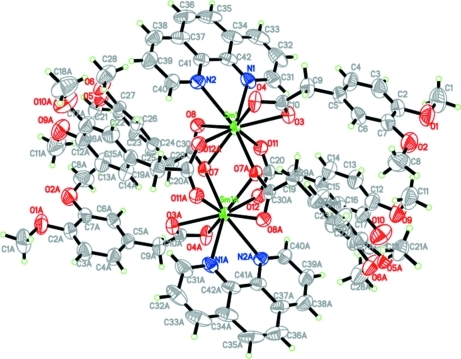
The molecular structure of the title complex, showing the atom-labelling scheme. Displacement ellipsoids are drawn at the 50% probability level.

## supplementary crystallographic information

### Comment

In recent years, there has been an increasing interest in the coordination chemistry due to the increased recognition of it's importance in magnetism (Yao, *et al.*, 2008; Fang, *et al.*, 2006; Wang, *et al.*, 2008) and antibacterial properties; we have worked in it before (Li, *et al.*, 2008; Liu, *et al.*, 2010). Here we report the crystal structure of a new samarium^III^ complex with the ligand 3,4-dimethoxylphenyl acetic acid. The title compound consists of two central samarium^III^ atoms coordinated with six homoveratric acid molecules and two phen molecules. In the bicentric structure, the DMPA ligands coordinate in the bis-chelate, bridging and bridging tridentate modes. Each samarium atom is nine coordinated from seven O atoms from five DMPA molecules and two N atoms from a phen molecule (Fig.1). The samarium^III^ atom is in a distorted capped square prismatic environment. The Sm—O bond lengths range from 2.397 (2) Å to 2.563 (2) Å and the Sm—N distances range from 2.591 (3) Å to 2.656 (3) Å. Intermolecular interactions (see Table 1) are C—H···O hydrogen bonds and weak π···π aromatic interactions from phen molecules and aromatic rings of the *L* ligands.

### Experimental

All reagents and solvents used were of commercially available quality and without purified before using. The title compound was obtained by adding Sm_2_O_3_(0.5 mmol), homoveratric acid (3 mmol) phen (1 mmol) dissolved in 30 ml water, sealed in a 50 ml stainless steel reactor and kept three days at temperature of 433 K. Then the reactor was cooled to room temperature at a speed of 5 K per hour. After filtration of the solution and washing the deposition with ethanol, colorless crystals were obtained.

### Refinement

The structure was solved by direct methods and successive Fourier difference synthesis. The H atoms bonded to C atoms were positioned geometrically and refined using a riding model [aliphatic C—H =0.96 Å (*U*_iso_(H) = 1.5*U*_eq_(C)), aromatic C—H = 0.93 Å (*U*_iso_(H) = 1.2*U*_eq_(C))].

### Crystal data

**Table:**

[Sm_2_(C_10_H_11_O_4_)_6_(C_12_H_8_N_2_)_2_]	*Z* = 1
*M**_r_* = 1832.26	*F*(000) = 930
Triclinic, *P*1	*D*_x_ = 1.557 Mg m^−^^3^
Hall symbol: -p 1	Mo *K*α radiation, λ = 0.71073 Å
*a* = 12.3696 (1) Å	Cell parameters from 9931 reflections
*b* = 12.4344 (1) Å	θ = 1.4–27.6°
*c* = 14.7467 (1) Å	µ = 1.57 mm^−^^1^
α = 90.641 (1)°	*T* = 296 K
β = 103.492 (1)°	Block, colourless
γ = 116.648 (1)°	0.35 × 0.15 × 0.09 mm
*V* = 1953.71 (3) Å^3^	

### Data collection

**Table:**

Bruker APEXII area-detector diffractometer	9023 independent reflections
Radiation source: fine-focus sealed tube	7540 reflections with *I* > 2σ(*I*)
graphite	*R*_int_ = 0.032
ω scans	θ_max_ = 27.6°, θ_min_ = 1.4°
Absorption correction: multi-scan (*SADABS*; Bruker, 2009	*h* = −16→16
*T*_min_ = 0.757, *T*_max_ = 0.874	*k* = −15→16
30687 measured reflections	*l* = −19→18

### Refinement

**Table:**

Refinement on *F*^2^	Primary atom site location: structure-invariant direct methods
Least-squares matrix: full	Secondary atom site location: difference Fourier map
*R*[*F*^2^ > 2σ(*F*^2^)] = 0.031	Hydrogen site location: inferred from neighbouring sites
*wR*(*F*^2^) = 0.102	H-atom parameters constrained
*S* = 0.76	*w* = 1/[σ^2^(*F*_o_^2^) + (0.1*P*)^2^] where *P* = (*F*_o_^2^ + 2*F*_c_^2^)/3
9023 reflections	(Δ/σ)_max_ = 0.001
514 parameters	Δρ_max_ = 0.80 e Å^−^^3^
0 restraints	Δρ_min_ = −0.49 e Å^−^^3^

### Special details

**Table:**

Geometry. All e.s.d.'s (except the e.s.d. in the dihedral angle between two l.s. planes) are estimated using the full covariance matrix. The cell e.s.d.'s are taken into account individually in the estimation of e.s.d.'s in distances, angles and torsion angles; correlations between e.s.d.'s in cell parameters are only used when they are defined by crystal symmetry. An approximate (isotropic) treatment of cell e.s.d.'s is used for estimating e.s.d.'s involving l.s. planes.
Refinement. Refinement of *F*^2^ against ALL reflections. The weighted *R*-factor *wR* and goodness of fit *S* are based on *F*^2^, conventional *R*-factors *R* are based on *F*, with *F* set to zero for negative *F*^2^. The threshold expression of *F*^2^ > σ(*F*^2^) is used only for calculating *R*-factors(gt) *etc*. and is not relevant to the choice of reflections for refinement. *R*-factors based on *F*^2^ are statistically about twice as large as those based on *F*, and *R*- factors based on ALL data will be even larger.

### Fractional atomic coordinates and isotropic or equivalent isotropic displacement parameters (Å^2^)

**Table:**

	*x*	*y*	*z*	*U*_iso_*/*U*_eq_	
Sm1	−0.181987 (12)	0.388581 (13)	0.966794 (9)	0.03674 (7)	
N1	−0.3981 (3)	0.3465 (3)	0.9898 (2)	0.0492 (6)	
O1	−0.2349 (3)	−0.0181 (3)	1.4333 (2)	0.0785 (9)	
C1	−0.3117 (6)	−0.0058 (5)	1.4856 (3)	0.0959 (16)	
H1A	−0.2851	−0.0180	1.5495	0.144*	
H1B	−0.3974	−0.0651	1.4584	0.144*	
H1C	−0.3049	0.0742	1.4846	0.144*	
N2	−0.3687 (2)	0.3332 (2)	0.81292 (19)	0.0438 (6)	
C2	−0.2610 (4)	−0.0030 (3)	1.3402 (3)	0.0565 (9)	
O2	−0.1033 (3)	−0.0586 (3)	1.3409 (2)	0.0763 (8)	
O3	−0.2346 (2)	0.24081 (19)	1.08276 (15)	0.0456 (5)	
C3	−0.3491 (4)	0.0293 (4)	1.2953 (3)	0.0646 (10)	
H3A	−0.3960	0.0460	1.3289	0.078*	
O4	−0.2685 (3)	0.1685 (2)	0.93737 (15)	0.0560 (6)	
C4	−0.3697 (4)	0.0374 (4)	1.1995 (3)	0.0603 (9)	
H4A	−0.4309	0.0585	1.1693	0.072*	
O5	−0.3659 (2)	0.9419 (2)	0.69521 (16)	0.0506 (6)	
C5	−0.3001 (3)	0.0143 (3)	1.1488 (2)	0.0496 (8)	
O6	−0.3882 (3)	0.7370 (2)	0.63142 (19)	0.0662 (8)	
C6	−0.2089 (3)	−0.0154 (3)	1.1954 (2)	0.0513 (8)	
H6A	−0.1600	−0.0293	1.1622	0.062*	
O7	−0.01393 (19)	0.6002 (2)	0.95385 (15)	0.0430 (5)	
C7	−0.1884 (3)	−0.0251 (3)	1.2902 (3)	0.0537 (8)	
O8	−0.20791 (19)	0.5702 (2)	0.91159 (16)	0.0454 (5)	
C8	−0.0163 (4)	−0.0667 (5)	1.2970 (4)	0.0873 (15)	
H8A	0.0377	−0.0907	1.3399	0.131*	
H8B	0.0332	0.0109	1.2795	0.131*	
H8C	−0.0609	−0.1259	1.2418	0.131*	
C9	−0.3203 (4)	0.0245 (3)	1.0452 (3)	0.0583 (9)	
H9A	−0.4096	−0.0193	1.0148	0.070*	
H9B	−0.2801	−0.0149	1.0191	0.070*	
O9	0.1593 (3)	0.3222 (3)	1.5044 (2)	0.0808 (9)	
C10	−0.2708 (3)	0.1535 (3)	1.0213 (2)	0.0416 (7)	
O10	0.2503 (4)	0.5480 (3)	1.5625 (3)	0.0970 (12)	
O11	−0.1156 (2)	0.5174 (2)	1.11307 (15)	0.0463 (5)	
C11	0.1067 (6)	0.1954 (5)	1.4761 (4)	0.0946 (17)	
H11A	0.1456	0.1608	1.5224	0.142*	
H11B	0.1207	0.1821	1.4166	0.142*	
H11C	0.0181	0.1576	1.4702	0.142*	
C12	0.1146 (4)	0.3845 (4)	1.4466 (3)	0.0630 (10)	
C13	0.0240 (4)	0.3342 (4)	1.3610 (3)	0.0657 (10)	
H13A	−0.0094	0.2519	1.3405	0.079*	
C14	−0.0176 (4)	0.4051 (4)	1.3056 (3)	0.0633 (10)	
H14A	−0.0782	0.3702	1.2485	0.076*	
C15	0.0310 (3)	0.5265 (3)	1.3353 (2)	0.0483 (7)	
C16	0.1196 (4)	0.5780 (4)	1.4207 (3)	0.0595 (9)	
H16A	0.1510	0.6599	1.4414	0.071*	
C17	0.1624 (4)	0.5066 (4)	1.4765 (3)	0.0631 (10)	
C18	0.2931 (6)	0.6660 (5)	1.6030 (4)	0.108 (2)	
H18A	0.3537	0.6822	1.6622	0.161*	
H18B	0.2238	0.6758	1.6128	0.161*	
H18C	0.3314	0.7218	1.5618	0.161*	
C19	−0.0160 (4)	0.6028 (4)	1.2739 (2)	0.0564 (9)	
H19A	−0.1011	0.5814	1.2755	0.068*	
H19B	0.0364	0.6881	1.2984	0.068*	
C20	−0.0131 (3)	0.5816 (3)	1.1727 (2)	0.0437 (7)	
C21	−0.3324 (4)	1.0664 (3)	0.7158 (3)	0.0589 (9)	
H21A	−0.3903	1.0856	0.6724	0.088*	
H21B	−0.2490	1.1157	0.7099	0.088*	
H21C	−0.3351	1.0822	0.7788	0.088*	
C22	−0.2914 (3)	0.9014 (3)	0.7514 (2)	0.0414 (6)	
C23	−0.2108 (3)	0.9586 (3)	0.8370 (2)	0.0523 (8)	
H23A	−0.2061	1.0299	0.8624	0.063*	
C24	−0.1356 (4)	0.9118 (3)	0.8867 (3)	0.0580 (9)	
H24A	−0.0827	0.9513	0.9458	0.070*	
C25	−0.1372 (3)	0.8079 (3)	0.8506 (2)	0.0461 (7)	
C26	−0.2253 (3)	0.7462 (3)	0.7652 (2)	0.0430 (7)	
H26A	−0.2328	0.6729	0.7412	0.052*	
C27	−0.3006 (3)	0.7910 (3)	0.7161 (2)	0.0404 (6)	
C28	−0.4229 (5)	0.6147 (4)	0.6042 (3)	0.0784 (14)	
H28A	−0.4842	0.5867	0.5443	0.118*	
H28B	−0.4579	0.5666	0.6503	0.118*	
H28C	−0.3502	0.6075	0.5996	0.118*	
C29	−0.0443 (3)	0.7655 (3)	0.8985 (3)	0.0548 (9)	
H29A	0.0074	0.8200	0.9563	0.066*	
H29B	0.0104	0.7743	0.8584	0.066*	
C30	−0.0952 (3)	0.6370 (3)	0.9225 (2)	0.0368 (6)	
C31	−0.4152 (4)	0.3464 (4)	1.0747 (3)	0.0639 (10)	
H31A	−0.3528	0.3489	1.1255	0.077*	
C32	−0.5237 (4)	0.3425 (4)	1.0913 (4)	0.0748 (13)	
H32A	−0.5339	0.3406	1.1519	0.090*	
C33	−0.6140 (4)	0.3416 (4)	1.0176 (4)	0.0772 (14)	
H33A	−0.6856	0.3409	1.0279	0.093*	
C34	−0.5995 (3)	0.3415 (3)	0.9269 (3)	0.0621 (10)	
C35	−0.6890 (3)	0.3424 (4)	0.8447 (4)	0.0786 (15)	
H35A	−0.7598	0.3461	0.8525	0.094*	
C36	−0.6745 (4)	0.3382 (4)	0.7580 (4)	0.0728 (13)	
H36A	−0.7339	0.3402	0.7070	0.087*	
C37	−0.5682 (3)	0.3307 (3)	0.7439 (3)	0.0567 (10)	
C38	−0.5517 (4)	0.3200 (3)	0.6545 (3)	0.0631 (10)	
H38A	−0.6130	0.3136	0.6013	0.076*	
C39	−0.4458 (4)	0.3189 (3)	0.6458 (3)	0.0611 (9)	
H39A	−0.4329	0.3131	0.5867	0.073*	
C40	−0.3554 (3)	0.3268 (3)	0.7272 (2)	0.0513 (8)	
H40A	−0.2822	0.3275	0.7202	0.062*	
C41	−0.4741 (3)	0.3349 (3)	0.8221 (3)	0.0447 (7)	
C42	−0.4901 (3)	0.3408 (3)	0.9152 (3)	0.0484 (8)	
O12	0.0936 (2)	0.6289 (2)	1.15752 (15)	0.0510 (5)	

### Atomic displacement parameters (Å^2^)

**Table:**

	*U*^11^	*U*^22^	*U*^33^	*U*^12^	*U*^13^	*U*^23^
Sm1	0.03906 (10)	0.05158 (12)	0.03811 (10)	0.03329 (8)	0.01703 (7)	0.01837 (7)
N1	0.0488 (15)	0.0539 (16)	0.0651 (17)	0.0325 (12)	0.0322 (13)	0.0199 (13)
O1	0.090 (2)	0.111 (3)	0.0478 (15)	0.0536 (19)	0.0266 (14)	0.0320 (15)
C1	0.131 (5)	0.122 (4)	0.058 (3)	0.069 (4)	0.045 (3)	0.027 (3)
N2	0.0387 (13)	0.0473 (14)	0.0554 (15)	0.0284 (11)	0.0124 (11)	0.0135 (12)
C2	0.061 (2)	0.059 (2)	0.0487 (19)	0.0244 (17)	0.0201 (16)	0.0199 (16)
O2	0.0709 (18)	0.102 (2)	0.0763 (19)	0.0536 (17)	0.0251 (15)	0.0396 (16)
O3	0.0597 (13)	0.0469 (12)	0.0419 (11)	0.0310 (10)	0.0213 (10)	0.0136 (9)
C3	0.074 (2)	0.080 (3)	0.059 (2)	0.045 (2)	0.0318 (19)	0.0208 (19)
O4	0.0879 (18)	0.0600 (14)	0.0364 (11)	0.0461 (13)	0.0202 (11)	0.0164 (10)
C4	0.068 (2)	0.075 (2)	0.053 (2)	0.045 (2)	0.0181 (17)	0.0225 (18)
O5	0.0597 (14)	0.0521 (13)	0.0489 (12)	0.0389 (11)	0.0031 (10)	0.0108 (10)
C5	0.0547 (18)	0.0403 (17)	0.0520 (18)	0.0197 (14)	0.0154 (15)	0.0166 (14)
O6	0.0804 (18)	0.0497 (14)	0.0550 (15)	0.0353 (13)	−0.0162 (13)	−0.0021 (11)
C6	0.0575 (19)	0.0453 (18)	0.0577 (19)	0.0243 (15)	0.0254 (16)	0.0179 (15)
O7	0.0450 (11)	0.0622 (13)	0.0460 (11)	0.0416 (10)	0.0191 (9)	0.0239 (10)
C7	0.0521 (19)	0.053 (2)	0.058 (2)	0.0245 (15)	0.0172 (16)	0.0228 (16)
O8	0.0412 (11)	0.0519 (12)	0.0590 (13)	0.0337 (10)	0.0155 (10)	0.0204 (10)
C8	0.063 (3)	0.091 (3)	0.121 (4)	0.047 (3)	0.024 (3)	0.032 (3)
C9	0.080 (3)	0.054 (2)	0.0434 (18)	0.0308 (19)	0.0204 (17)	0.0118 (15)
O9	0.096 (2)	0.082 (2)	0.0684 (18)	0.0536 (18)	0.0025 (16)	0.0195 (15)
C10	0.0421 (15)	0.0515 (18)	0.0416 (16)	0.0304 (14)	0.0113 (12)	0.0132 (13)
O10	0.097 (3)	0.092 (2)	0.086 (2)	0.047 (2)	−0.010 (2)	−0.0098 (19)
O11	0.0458 (12)	0.0621 (14)	0.0460 (12)	0.0346 (11)	0.0188 (10)	0.0139 (10)
C11	0.111 (4)	0.081 (3)	0.090 (4)	0.050 (3)	0.012 (3)	0.033 (3)
C12	0.067 (2)	0.076 (3)	0.054 (2)	0.040 (2)	0.0149 (17)	0.0184 (18)
C13	0.080 (3)	0.063 (2)	0.053 (2)	0.035 (2)	0.0121 (18)	0.0099 (17)
C14	0.074 (2)	0.072 (3)	0.0454 (18)	0.038 (2)	0.0099 (17)	0.0114 (17)
C15	0.0489 (17)	0.064 (2)	0.0435 (16)	0.0315 (16)	0.0216 (14)	0.0143 (14)
C16	0.057 (2)	0.063 (2)	0.059 (2)	0.0290 (17)	0.0156 (17)	0.0093 (17)
C17	0.058 (2)	0.082 (3)	0.054 (2)	0.039 (2)	0.0096 (17)	0.0145 (19)
C18	0.095 (4)	0.114 (5)	0.085 (4)	0.043 (3)	−0.012 (3)	−0.037 (3)
C19	0.067 (2)	0.075 (2)	0.0487 (18)	0.0458 (19)	0.0264 (16)	0.0152 (16)
C20	0.0490 (17)	0.0607 (19)	0.0416 (16)	0.0387 (15)	0.0196 (13)	0.0193 (14)
C21	0.077 (2)	0.061 (2)	0.058 (2)	0.051 (2)	0.0119 (18)	0.0148 (16)
C22	0.0420 (15)	0.0451 (16)	0.0468 (16)	0.0279 (13)	0.0129 (13)	0.0136 (13)
C23	0.065 (2)	0.0527 (19)	0.0485 (17)	0.0414 (17)	0.0032 (15)	0.0003 (14)
C24	0.066 (2)	0.057 (2)	0.0491 (19)	0.0392 (18)	−0.0101 (16)	−0.0028 (15)
C25	0.0429 (16)	0.0466 (17)	0.0570 (18)	0.0287 (14)	0.0105 (14)	0.0176 (14)
C26	0.0544 (17)	0.0399 (16)	0.0445 (16)	0.0301 (14)	0.0132 (13)	0.0101 (12)
C27	0.0438 (15)	0.0379 (15)	0.0408 (15)	0.0217 (12)	0.0079 (12)	0.0091 (12)
C28	0.091 (3)	0.051 (2)	0.072 (3)	0.032 (2)	−0.012 (2)	−0.0083 (19)
C29	0.0419 (17)	0.051 (2)	0.074 (2)	0.0295 (15)	0.0028 (16)	0.0179 (18)
C30	0.0440 (15)	0.0483 (16)	0.0352 (14)	0.0344 (13)	0.0134 (12)	0.0139 (12)
C31	0.063 (2)	0.071 (3)	0.077 (3)	0.037 (2)	0.042 (2)	0.021 (2)
C32	0.080 (3)	0.065 (2)	0.102 (3)	0.033 (2)	0.064 (3)	0.017 (2)
C33	0.051 (2)	0.057 (2)	0.139 (4)	0.0272 (18)	0.049 (3)	0.002 (2)
C34	0.0412 (17)	0.0409 (18)	0.111 (3)	0.0208 (14)	0.0294 (19)	0.0022 (19)
C35	0.0306 (17)	0.059 (2)	0.148 (5)	0.0268 (16)	0.014 (2)	0.003 (3)
C36	0.0404 (19)	0.058 (2)	0.112 (4)	0.0266 (17)	−0.001 (2)	0.001 (2)
C37	0.0363 (16)	0.0342 (16)	0.092 (3)	0.0176 (13)	0.0016 (17)	0.0106 (16)
C38	0.055 (2)	0.053 (2)	0.069 (2)	0.0244 (17)	−0.0029 (18)	0.0116 (18)
C39	0.067 (2)	0.060 (2)	0.057 (2)	0.0346 (19)	0.0060 (17)	0.0135 (17)
C40	0.0532 (19)	0.0536 (19)	0.0545 (19)	0.0321 (16)	0.0118 (15)	0.0157 (15)
C41	0.0350 (14)	0.0347 (15)	0.067 (2)	0.0198 (12)	0.0107 (14)	0.0103 (14)
C42	0.0343 (14)	0.0346 (15)	0.086 (2)	0.0215 (12)	0.0210 (15)	0.0099 (15)
O12	0.0462 (12)	0.0756 (15)	0.0424 (11)	0.0352 (11)	0.0174 (9)	0.0122 (11)

### Geometric parameters (Å, °)

**Table:**

Sm1—O7^i^	2.373 (2)	C12—C13	1.391 (5)
Sm1—O12^i^	2.397 (2)	C13—C14	1.392 (6)
Sm1—O11	2.410 (2)	C13—H13A	0.9300
Sm1—O4	2.440 (2)	C14—C15	1.372 (5)
Sm1—O3	2.498 (2)	C14—H14A	0.9300
Sm1—O8	2.537 (2)	C15—C16	1.380 (5)
Sm1—O7	2.564 (2)	C15—C19	1.525 (5)
Sm1—N1	2.590 (3)	C16—C17	1.407 (6)
Sm1—N2	2.655 (3)	C16—H16A	0.9300
Sm1—C10	2.825 (3)	C18—H18A	0.9600
Sm1—C30	2.919 (3)	C18—H18B	0.9600
Sm1—Sm1^i^	3.9495 (3)	C18—H18C	0.9600
N1—C31	1.318 (5)	C19—C20	1.524 (4)
N1—C42	1.360 (5)	C19—H19A	0.9700
O1—C2	1.370 (4)	C19—H19B	0.9700
O1—C1	1.411 (6)	C20—O12	1.257 (4)
C1—H1A	0.9600	C21—H21A	0.9600
C1—H1B	0.9600	C21—H21B	0.9600
C1—H1C	0.9600	C21—H21C	0.9600
N2—C40	1.319 (4)	C22—C23	1.360 (5)
N2—C41	1.352 (4)	C22—C27	1.409 (4)
C2—C3	1.360 (6)	C23—C24	1.386 (5)
C2—C7	1.396 (5)	C23—H23A	0.9300
O2—C7	1.369 (4)	C24—C25	1.381 (5)
O2—C8	1.418 (6)	C24—H24A	0.9300
O3—C10	1.246 (4)	C25—C26	1.397 (4)
C3—C4	1.388 (5)	C25—C29	1.507 (4)
C3—H3A	0.9300	C26—C27	1.364 (4)
O4—C10	1.258 (4)	C26—H26A	0.9300
C4—C5	1.379 (5)	C28—H28A	0.9600
C4—H4A	0.9300	C28—H28B	0.9600
O5—C22	1.363 (4)	C28—H28C	0.9600
O5—C21	1.420 (4)	C29—C30	1.512 (5)
C5—C6	1.379 (5)	C29—H29A	0.9700
C5—C9	1.506 (5)	C29—H29B	0.9700
O6—C27	1.372 (4)	C31—C32	1.399 (6)
O6—C28	1.406 (5)	C31—H31A	0.9300
C6—C7	1.378 (5)	C32—C33	1.361 (7)
C6—H6A	0.9300	C32—H32A	0.9300
O7—C30	1.279 (3)	C33—C34	1.390 (7)
O7—Sm1^i^	2.3731 (19)	C33—H33A	0.9300
O8—C30	1.230 (4)	C34—C42	1.407 (4)
C8—H8A	0.9600	C34—C35	1.443 (7)
C8—H8B	0.9600	C35—C36	1.336 (7)
C8—H8C	0.9600	C35—H35A	0.9300
C9—C10	1.518 (5)	C36—C37	1.421 (6)
C9—H9A	0.9700	C36—H36A	0.9300
C9—H9B	0.9700	C37—C38	1.394 (6)
O9—C12	1.345 (5)	C37—C41	1.415 (5)
O9—C11	1.422 (6)	C38—C39	1.351 (6)
O10—C17	1.377 (5)	C38—H38A	0.9300
O10—C18	1.387 (6)	C39—C40	1.405 (5)
O11—C20	1.255 (4)	C39—H39A	0.9300
C11—H11A	0.9600	C40—H40A	0.9300
C11—H11B	0.9600	C41—C42	1.438 (5)
C11—H11C	0.9600	O12—Sm1^i^	2.397 (2)
C12—C17	1.381 (6)		
			
O7^i^—Sm1—O12^i^	75.89 (7)	O4—C10—Sm1	59.49 (17)
O7^i^—Sm1—O11	75.04 (7)	C9—C10—Sm1	177.0 (2)
O12^i^—Sm1—O11	137.69 (8)	C17—O10—C18	118.7 (4)
O7^i^—Sm1—O4	88.68 (8)	C20—O11—Sm1	135.8 (2)
O12^i^—Sm1—O4	79.61 (8)	O9—C11—H11A	109.5
O11—Sm1—O4	129.32 (7)	O9—C11—H11B	109.5
O7^i^—Sm1—O3	75.96 (7)	H11A—C11—H11B	109.5
O12^i^—Sm1—O3	124.10 (8)	O9—C11—H11C	109.5
O11—Sm1—O3	76.85 (7)	H11A—C11—H11C	109.5
O4—Sm1—O3	52.55 (7)	H11B—C11—H11C	109.5
O7^i^—Sm1—O8	123.58 (7)	O9—C12—C17	116.6 (4)
O12^i^—Sm1—O8	93.53 (8)	O9—C12—C13	124.7 (4)
O11—Sm1—O8	77.97 (7)	C17—C12—C13	118.8 (4)
O4—Sm1—O8	144.58 (8)	C12—C13—C14	121.0 (4)
O3—Sm1—O8	142.00 (7)	C12—C13—H13A	119.5
O7^i^—Sm1—O7	73.80 (7)	C14—C13—H13A	119.5
O12^i^—Sm1—O7	71.45 (8)	C15—C14—C13	119.9 (4)
O11—Sm1—O7	71.33 (7)	C15—C14—H14A	120.1
O4—Sm1—O7	148.95 (8)	C13—C14—H14A	120.1
O3—Sm1—O7	140.55 (7)	C14—C15—C16	120.1 (4)
O8—Sm1—O7	50.76 (6)	C14—C15—C19	119.2 (3)
O7^i^—Sm1—N1	142.48 (8)	C16—C15—C19	120.7 (4)
O12^i^—Sm1—N1	138.87 (9)	C15—C16—C17	120.1 (4)
O11—Sm1—N1	79.86 (8)	C15—C16—H16A	120.0
O4—Sm1—N1	86.07 (9)	C17—C16—H16A	120.0
O3—Sm1—N1	71.45 (8)	O10—C17—C12	114.7 (4)
O8—Sm1—N1	76.46 (8)	O10—C17—C16	125.1 (4)
O7—Sm1—N1	123.34 (8)	C12—C17—C16	120.1 (4)
O7^i^—Sm1—N2	151.34 (8)	O10—C18—H18A	109.5
O12^i^—Sm1—N2	76.66 (8)	O10—C18—H18B	109.5
O11—Sm1—N2	132.54 (8)	H18A—C18—H18B	109.5
O4—Sm1—N2	78.41 (8)	O10—C18—H18C	109.5
O3—Sm1—N2	113.77 (8)	H18A—C18—H18C	109.5
O8—Sm1—N2	66.22 (7)	H18B—C18—H18C	109.5
O7—Sm1—N2	104.89 (7)	C20—C19—C15	110.5 (3)
N1—Sm1—N2	62.70 (9)	C20—C19—H19A	109.5
O7^i^—Sm1—C10	81.44 (8)	C15—C19—H19A	109.5
O12^i^—Sm1—C10	102.32 (9)	C20—C19—H19B	109.5
O11—Sm1—C10	102.99 (9)	C15—C19—H19B	109.5
O4—Sm1—C10	26.38 (8)	H19A—C19—H19B	108.1
O3—Sm1—C10	26.17 (8)	O11—C20—O12	126.1 (3)
O8—Sm1—C10	153.37 (8)	O11—C20—C19	117.7 (3)
O7—Sm1—C10	155.23 (8)	O12—C20—C19	116.1 (3)
N1—Sm1—C10	77.54 (9)	O5—C21—H21A	109.5
N2—Sm1—C10	96.56 (9)	O5—C21—H21B	109.5
O7^i^—Sm1—C30	99.31 (8)	H21A—C21—H21B	109.5
O12^i^—Sm1—C30	81.97 (8)	O5—C21—H21C	109.5
O11—Sm1—C30	73.21 (8)	H21A—C21—H21C	109.5
O4—Sm1—C30	157.47 (8)	H21B—C21—H21C	109.5
O3—Sm1—C30	149.86 (8)	C23—C22—O5	125.4 (3)
O8—Sm1—C30	24.81 (7)	C23—C22—C27	118.7 (3)
O7—Sm1—C30	25.95 (7)	O5—C22—C27	116.0 (3)
N1—Sm1—C30	99.58 (8)	C22—C23—C24	120.6 (3)
N2—Sm1—C30	84.83 (8)	C22—C23—H23A	119.7
C10—Sm1—C30	175.68 (8)	C24—C23—H23A	119.7
O7^i^—Sm1—Sm1^i^	38.56 (5)	C25—C24—C23	121.6 (3)
O12^i^—Sm1—Sm1^i^	69.33 (5)	C25—C24—H24A	119.2
O11—Sm1—Sm1^i^	68.72 (5)	C23—C24—H24A	119.2
O4—Sm1—Sm1^i^	122.84 (7)	C24—C25—C26	117.2 (3)
O3—Sm1—Sm1^i^	110.82 (5)	C24—C25—C29	121.7 (3)
O8—Sm1—Sm1^i^	85.51 (5)	C26—C25—C29	121.0 (3)
O7—Sm1—Sm1^i^	35.24 (4)	C27—C26—C25	121.5 (3)
N1—Sm1—Sm1^i^	146.45 (7)	C27—C26—H26A	119.3
N2—Sm1—Sm1^i^	133.95 (6)	C25—C26—H26A	119.3
C10—Sm1—Sm1^i^	119.99 (6)	C26—C27—O6	125.1 (3)
C30—Sm1—Sm1^i^	60.89 (6)	C26—C27—C22	120.2 (3)
C31—N1—C42	118.9 (3)	O6—C27—C22	114.6 (3)
C31—N1—Sm1	120.7 (3)	O6—C28—H28A	109.5
C42—N1—Sm1	119.8 (2)	O6—C28—H28B	109.5
C2—O1—C1	116.7 (4)	H28A—C28—H28B	109.5
O1—C1—H1A	109.5	O6—C28—H28C	109.5
O1—C1—H1B	109.5	H28A—C28—H28C	109.5
H1A—C1—H1B	109.5	H28B—C28—H28C	109.5
O1—C1—H1C	109.5	C25—C29—C30	117.9 (3)
H1A—C1—H1C	109.5	C25—C29—H29A	108.0
H1B—C1—H1C	109.5	C30—C29—H29A	108.0
C40—N2—C41	117.6 (3)	C25—C29—H29B	108.0
C40—N2—Sm1	123.0 (2)	C30—C29—H29B	108.0
C41—N2—Sm1	118.2 (2)	H29A—C29—H29B	107.0
C3—C2—O1	126.0 (4)	O8—C30—O7	121.2 (3)
C3—C2—C7	119.8 (3)	O8—C30—C29	122.8 (3)
O1—C2—C7	114.2 (4)	O7—C30—C29	116.0 (3)
C7—O2—C8	117.9 (3)	O8—C30—Sm1	59.92 (16)
C10—O3—Sm1	91.70 (18)	O7—C30—Sm1	61.30 (16)
C2—C3—C4	120.5 (4)	C29—C30—Sm1	177.0 (2)
C2—C3—H3A	119.8	N1—C31—C32	122.4 (4)
C4—C3—H3A	119.8	N1—C31—H31A	118.8
C10—O4—Sm1	94.1 (2)	C32—C31—H31A	118.8
C5—C4—C3	120.6 (4)	C33—C32—C31	119.1 (4)
C5—C4—H4A	119.7	C33—C32—H32A	120.4
C3—C4—H4A	119.7	C31—C32—H32A	120.4
C22—O5—C21	116.1 (3)	C32—C33—C34	120.2 (4)
C6—C5—C4	118.5 (3)	C32—C33—H33A	119.9
C6—C5—C9	120.4 (4)	C34—C33—H33A	119.9
C4—C5—C9	121.0 (4)	C33—C34—C42	117.5 (4)
C27—O6—C28	116.8 (3)	C33—C34—C35	124.2 (4)
C7—C6—C5	121.5 (4)	C42—C34—C35	118.3 (4)
C7—C6—H6A	119.2	C36—C35—C34	122.8 (4)
C5—C6—H6A	119.2	C36—C35—H35A	118.6
C30—O7—Sm1^i^	158.2 (2)	C34—C35—H35A	118.6
C30—O7—Sm1	92.76 (18)	C35—C36—C37	119.8 (4)
Sm1^i^—O7—Sm1	106.20 (7)	C35—C36—H36A	120.1
O2—C7—C6	125.1 (4)	C37—C36—H36A	120.1
O2—C7—C2	115.7 (3)	C38—C37—C41	117.7 (3)
C6—C7—C2	119.1 (3)	C38—C37—C36	122.2 (4)
C30—O8—Sm1	95.27 (17)	C41—C37—C36	120.1 (4)
O2—C8—H8A	109.5	C39—C38—C37	119.5 (3)
O2—C8—H8B	109.5	C39—C38—H38A	120.3
H8A—C8—H8B	109.5	C37—C38—H38A	120.3
O2—C8—H8C	109.5	C38—C39—C40	119.2 (4)
H8A—C8—H8C	109.5	C38—C39—H39A	120.4
H8B—C8—H8C	109.5	C40—C39—H39A	120.4
C5—C9—C10	114.8 (3)	N2—C40—C39	123.5 (4)
C5—C9—H9A	108.6	N2—C40—H40A	118.3
C10—C9—H9A	108.6	C39—C40—H40A	118.3
C5—C9—H9B	108.6	N2—C41—C37	122.5 (3)
C10—C9—H9B	108.6	N2—C41—C42	118.0 (3)
H9A—C9—H9B	107.5	C37—C41—C42	119.5 (3)
C12—O9—C11	116.9 (4)	N1—C42—C34	121.8 (4)
O3—C10—O4	121.6 (3)	N1—C42—C41	118.9 (3)
O3—C10—C9	120.9 (3)	C34—C42—C41	119.3 (3)
O4—C10—C9	117.5 (3)	C20—O12—Sm1^i^	136.8 (2)
O3—C10—Sm1	62.13 (17)		
			
O7^i^—Sm1—N1—C31	14.7 (4)	O7^i^—Sm1—C10—O4	−104.6 (2)
O12^i^—Sm1—N1—C31	166.7 (3)	O12^i^—Sm1—C10—O4	−31.1 (2)
O11—Sm1—N1—C31	−33.7 (3)	O11—Sm1—C10—O4	−176.9 (2)
O4—Sm1—N1—C31	97.4 (3)	O3—Sm1—C10—O4	179.9 (3)
O3—Sm1—N1—C31	45.7 (3)	O8—Sm1—C10—O4	94.0 (3)
O8—Sm1—N1—C31	−113.7 (3)	O7—Sm1—C10—O4	−103.4 (3)
O7—Sm1—N1—C31	−93.3 (3)	N1—Sm1—C10—O4	106.7 (2)
N2—Sm1—N1—C31	176.4 (3)	N2—Sm1—C10—O4	46.6 (2)
C10—Sm1—N1—C31	72.1 (3)	Sm1^i^—Sm1—C10—O4	−104.2 (2)
C30—Sm1—N1—C31	−104.6 (3)	O7^i^—Sm1—O11—C20	21.9 (3)
Sm1^i^—Sm1—N1—C31	−54.2 (3)	O12^i^—Sm1—O11—C20	−26.2 (3)
O7^i^—Sm1—N1—C42	−174.3 (2)	O4—Sm1—O11—C20	97.4 (3)
O12^i^—Sm1—N1—C42	−22.3 (3)	O3—Sm1—O11—C20	100.6 (3)
O11—Sm1—N1—C42	137.3 (2)	O8—Sm1—O11—C20	−108.1 (3)
O4—Sm1—N1—C42	−91.6 (2)	O7—Sm1—O11—C20	−55.7 (3)
O3—Sm1—N1—C42	−143.3 (2)	N1—Sm1—O11—C20	173.8 (3)
O8—Sm1—N1—C42	57.4 (2)	N2—Sm1—O11—C20	−149.0 (3)
O7—Sm1—N1—C42	77.8 (2)	C10—Sm1—O11—C20	99.2 (3)
N2—Sm1—N1—C42	−12.6 (2)	C30—Sm1—O11—C20	−82.9 (3)
C10—Sm1—N1—C42	−116.8 (2)	Sm1^i^—Sm1—O11—C20	−18.2 (3)
C30—Sm1—N1—C42	66.5 (2)	C11—O9—C12—C17	177.4 (4)
Sm1^i^—Sm1—N1—C42	116.8 (2)	C11—O9—C12—C13	−1.9 (7)
O7^i^—Sm1—N2—C40	−23.7 (3)	O9—C12—C13—C14	179.7 (4)
O12^i^—Sm1—N2—C40	−6.7 (3)	C17—C12—C13—C14	0.5 (7)
O11—Sm1—N2—C40	137.8 (2)	C12—C13—C14—C15	0.0 (7)
O4—Sm1—N2—C40	−88.6 (3)	C13—C14—C15—C16	−1.0 (6)
O3—Sm1—N2—C40	−128.4 (3)	C13—C14—C15—C19	−179.7 (3)
O8—Sm1—N2—C40	93.3 (3)	C14—C15—C16—C17	1.5 (6)
O7—Sm1—N2—C40	59.7 (3)	C19—C15—C16—C17	−179.8 (3)
N1—Sm1—N2—C40	179.8 (3)	C18—O10—C17—C12	−172.9 (5)
C10—Sm1—N2—C40	−107.8 (3)	C18—O10—C17—C16	6.1 (7)
C30—Sm1—N2—C40	76.3 (3)	O9—C12—C17—O10	−0.2 (6)
Sm1^i^—Sm1—N2—C40	36.2 (3)	C13—C12—C17—O10	179.1 (4)
O7^i^—Sm1—N2—C41	169.52 (19)	O9—C12—C17—C16	−179.3 (4)
O12^i^—Sm1—N2—C41	−173.5 (2)	C13—C12—C17—C16	0.0 (6)
O11—Sm1—N2—C41	−29.0 (3)	C15—C16—C17—O10	180.0 (4)
O4—Sm1—N2—C41	104.6 (2)	C15—C16—C17—C12	−1.0 (6)
O3—Sm1—N2—C41	64.9 (2)	C14—C15—C19—C20	−49.5 (5)
O8—Sm1—N2—C41	−73.5 (2)	C16—C15—C19—C20	131.7 (4)
O7—Sm1—N2—C41	−107.1 (2)	Sm1—O11—C20—O12	25.7 (5)
N1—Sm1—N2—C41	13.1 (2)	Sm1—O11—C20—C19	−152.8 (2)
C10—Sm1—N2—C41	85.4 (2)	C15—C19—C20—O11	105.0 (4)
C30—Sm1—N2—C41	−90.5 (2)	C15—C19—C20—O12	−73.7 (4)
Sm1^i^—Sm1—N2—C41	−130.55 (19)	C21—O5—C22—C23	17.6 (5)
C1—O1—C2—C3	3.9 (6)	C21—O5—C22—C27	−162.3 (3)
C1—O1—C2—C7	−175.5 (4)	O5—C22—C23—C24	−177.4 (3)
O7^i^—Sm1—O3—C10	−99.26 (19)	C27—C22—C23—C24	2.6 (5)
O12^i^—Sm1—O3—C10	−37.5 (2)	C22—C23—C24—C25	1.6 (6)
O11—Sm1—O3—C10	−176.9 (2)	C23—C24—C25—C26	−5.1 (6)
O4—Sm1—O3—C10	0.05 (18)	C23—C24—C25—C29	172.1 (4)
O8—Sm1—O3—C10	133.44 (19)	C24—C25—C26—C27	4.5 (5)
O7—Sm1—O3—C10	−140.09 (18)	C29—C25—C26—C27	−172.8 (3)
N1—Sm1—O3—C10	99.6 (2)	C25—C26—C27—O6	179.1 (3)
N2—Sm1—O3—C10	52.2 (2)	C25—C26—C27—C22	−0.4 (5)
C30—Sm1—O3—C10	176.40 (16)	C28—O6—C27—C26	15.4 (6)
Sm1^i^—Sm1—O3—C10	−116.03 (18)	C28—O6—C27—C22	−165.0 (4)
O1—C2—C3—C4	−177.7 (4)	C23—C22—C27—C26	−3.2 (5)
C7—C2—C3—C4	1.7 (6)	O5—C22—C27—C26	176.8 (3)
O7^i^—Sm1—O4—C10	73.2 (2)	C23—C22—C27—O6	177.2 (3)
O12^i^—Sm1—O4—C10	149.1 (2)	O5—C22—C27—O6	−2.8 (4)
O11—Sm1—O4—C10	3.9 (2)	C24—C25—C29—C30	125.6 (4)
O3—Sm1—O4—C10	−0.05 (18)	C26—C25—C29—C30	−57.3 (5)
O8—Sm1—O4—C10	−129.52 (19)	Sm1—O8—C30—O7	−0.6 (3)
O7—Sm1—O4—C10	127.8 (2)	Sm1—O8—C30—C29	178.4 (3)
N1—Sm1—O4—C10	−69.6 (2)	Sm1^i^—O7—C30—O8	−150.3 (4)
N2—Sm1—O4—C10	−132.5 (2)	Sm1—O7—C30—O8	0.6 (3)
C30—Sm1—O4—C10	−175.27 (18)	Sm1^i^—O7—C30—C29	30.7 (7)
Sm1^i^—Sm1—O4—C10	92.2 (2)	Sm1—O7—C30—C29	−178.5 (3)
C2—C3—C4—C5	−0.8 (6)	Sm1^i^—O7—C30—Sm1	−150.8 (5)
C3—C4—C5—C6	−0.8 (6)	C25—C29—C30—O8	−6.2 (5)
C3—C4—C5—C9	−179.2 (4)	C25—C29—C30—O7	172.8 (3)
C4—C5—C6—C7	1.5 (5)	O7^i^—Sm1—C30—O8	−168.86 (18)
C9—C5—C6—C7	179.9 (3)	O12^i^—Sm1—C30—O8	116.95 (19)
O7^i^—Sm1—O7—C30	−169.2 (2)	O11—Sm1—C30—O8	−97.69 (19)
O12^i^—Sm1—O7—C30	110.65 (18)	O4—Sm1—C30—O8	81.6 (3)
O11—Sm1—O7—C30	−89.85 (18)	O3—Sm1—C30—O8	−90.8 (2)
O4—Sm1—O7—C30	132.80 (19)	O7—Sm1—C30—O8	−179.4 (3)
O3—Sm1—O7—C30	−127.81 (17)	N1—Sm1—C30—O8	−21.4 (2)
O8—Sm1—O7—C30	−0.31 (16)	N2—Sm1—C30—O8	39.73 (19)
N1—Sm1—O7—C30	−26.3 (2)	Sm1^i^—Sm1—C30—O8	−172.3 (2)
N2—Sm1—O7—C30	40.59 (18)	O7^i^—Sm1—C30—O7	10.6 (2)
C10—Sm1—O7—C30	−170.29 (18)	O12^i^—Sm1—C30—O7	−63.63 (17)
Sm1^i^—Sm1—O7—C30	−169.2 (2)	O11—Sm1—C30—O7	81.73 (17)
O7^i^—Sm1—O7—Sm1^i^	0.0	O4—Sm1—C30—O7	−99.0 (3)
O12^i^—Sm1—O7—Sm1^i^	−80.20 (9)	O3—Sm1—C30—O7	88.6 (2)
O11—Sm1—O7—Sm1^i^	79.30 (9)	O8—Sm1—C30—O7	179.4 (3)
O4—Sm1—O7—Sm1^i^	−58.05 (17)	N1—Sm1—C30—O7	157.99 (17)
O3—Sm1—O7—Sm1^i^	41.34 (14)	N2—Sm1—C30—O7	−140.85 (17)
O8—Sm1—O7—Sm1^i^	168.84 (13)	Sm1^i^—Sm1—C30—O7	7.14 (14)
N1—Sm1—O7—Sm1^i^	142.90 (9)	C42—N1—C31—C32	−1.0 (6)
N2—Sm1—O7—Sm1^i^	−150.26 (9)	Sm1—N1—C31—C32	170.1 (3)
C10—Sm1—O7—Sm1^i^	−1.1 (2)	N1—C31—C32—C33	−1.5 (6)
C30—Sm1—O7—Sm1^i^	169.2 (2)	C31—C32—C33—C34	1.5 (6)
C8—O2—C7—C6	9.9 (6)	C32—C33—C34—C42	0.8 (6)
C8—O2—C7—C2	−172.0 (4)	C32—C33—C34—C35	−179.0 (4)
C5—C6—C7—O2	177.4 (3)	C33—C34—C35—C36	−177.7 (4)
C5—C6—C7—C2	−0.7 (5)	C42—C34—C35—C36	2.5 (6)
C3—C2—C7—O2	−179.2 (4)	C34—C35—C36—C37	0.9 (6)
O1—C2—C7—O2	0.2 (5)	C35—C36—C37—C38	176.8 (4)
C3—C2—C7—C6	−0.9 (6)	C35—C36—C37—C41	−3.8 (6)
O1—C2—C7—C6	178.5 (3)	C41—C37—C38—C39	−2.6 (5)
O7^i^—Sm1—O8—C30	13.2 (2)	C36—C37—C38—C39	176.9 (3)
O12^i^—Sm1—O8—C30	−62.17 (19)	C37—C38—C39—C40	1.0 (6)
O11—Sm1—O8—C30	75.94 (19)	C41—N2—C40—C39	−1.5 (5)
O4—Sm1—O8—C30	−139.16 (19)	Sm1—N2—C40—C39	−168.4 (3)
O3—Sm1—O8—C30	125.36 (19)	C38—C39—C40—N2	1.2 (6)
O7—Sm1—O8—C30	0.33 (17)	C40—N2—C41—C37	−0.3 (5)
N1—Sm1—O8—C30	158.3 (2)	Sm1—N2—C41—C37	167.2 (2)
N2—Sm1—O8—C30	−135.9 (2)	C40—N2—C41—C42	179.5 (3)
C10—Sm1—O8—C30	170.97 (18)	Sm1—N2—C41—C42	−13.0 (4)
Sm1^i^—Sm1—O8—C30	6.76 (18)	C38—C37—C41—N2	2.3 (5)
C6—C5—C9—C10	−105.2 (4)	C36—C37—C41—N2	−177.2 (3)
C4—C5—C9—C10	73.1 (5)	C38—C37—C41—C42	−177.4 (3)
Sm1—O3—C10—O4	−0.1 (3)	C36—C37—C41—C42	3.1 (5)
Sm1—O3—C10—C9	−179.7 (3)	C31—N1—C42—C34	3.5 (5)
Sm1—O4—C10—O3	0.1 (3)	Sm1—N1—C42—C34	−167.7 (2)
Sm1—O4—C10—C9	179.7 (3)	C31—N1—C42—C41	−176.9 (3)
C5—C9—C10—O3	−10.5 (5)	Sm1—N1—C42—C41	11.9 (4)
C5—C9—C10—O4	169.9 (3)	C33—C34—C42—N1	−3.4 (5)
O7^i^—Sm1—C10—O3	75.53 (19)	C35—C34—C42—N1	176.4 (3)
O12^i^—Sm1—C10—O3	148.96 (18)	C33—C34—C42—C41	177.1 (3)
O11—Sm1—C10—O3	3.1 (2)	C35—C34—C42—C41	−3.1 (5)
O4—Sm1—C10—O3	−179.9 (3)	N2—C41—C42—N1	1.1 (4)
O8—Sm1—C10—O3	−85.9 (3)	C37—C41—C42—N1	−179.1 (3)
O7—Sm1—C10—O3	76.6 (3)	N2—C41—C42—C34	−179.4 (3)
N1—Sm1—C10—O3	−73.20 (19)	C37—C41—C42—C34	0.4 (5)
N2—Sm1—C10—O3	−133.32 (19)	O11—C20—O12—Sm1^i^	−12.2 (6)
Sm1^i^—Sm1—C10—O3	75.88 (19)	C19—C20—O12—Sm1^i^	166.3 (2)

Symmetry codes: (i) −*x*, −*y*+1, −*z*+2.

### Hydrogen-bond geometry (Å, °)

**Table:**

*D*—H···*A*	*D*—H	H···*A*	*D*···*A*	*D*—H···*A*
C1—H1A···O5^ii^	0.96	2.54	3.323 (5)	139
C21—H21C···O4^iii^	0.96	2.36	3.274 (5)	160
C23—H23A···O4^iii^	0.93	2.52	3.404 (4)	160
C31—H31A···O3	0.93	2.55	3.040 (5)	113

Symmetry codes: (ii) *x*, *y*−1, *z*+1; (iii) *x*, *y*+1, *z*.
